# Efficacy of Three Kayviruses Against *Staphylococcus aureus* Strains Isolated from COVID-19 Patients

**DOI:** 10.3390/antibiotics14030257

**Published:** 2025-03-03

**Authors:** Lidia Piechowicz, Katarzyna Kosznik-Kwaśnicka, Natalia Kaźmierczak, Milena Grzenkowicz, Małgorzata Stasiłojć, Agnieszka Necel, Olesia Werbowy, Anna Pałubicka

**Affiliations:** 1 Department of Medical Microbiology, Faculty of Medicine, Medical University of Gdańsk, Dębowa 25, 80-204 Gdansk, Poland; nlubowska@gumed.edu.pl (N.K.); milena.grzenkowicz@gumed.edu.pl (M.G.); agnieszka.necel@gumed.edu.pl (A.N.); 2 Department of Microbiology, Faculty of Biology, University of Gdańsk, Wita Stwosza 59, 80-308 Gdansk, Poland; olesia.werbowy@ug.edu.pl; 3 Department of Cell Biology and Immunology, Intercollegiate Faculty of Biotechnology of University of Gdańsk and Medical University of Gdańsk, Dębinki 1, 80-211 Gdansk, Poland; malgorzata.stasilojc@gumed.edu.pl; 4 Specialist Hospital in Kościerzyna Sp. z o.o., Department of Laboratory and Microbiological Diagnostics, Kościerzyna, Alojzego Piechowskiego 36, 83-400 Koscierzyna, Poland; apalubicka@op.pl

**Keywords:** *Staphylococcus aureus*, COVID-19, bacteriophages, phage therapy

## Abstract

**Background/Objectives:** The viral pandemic caused by the SARS-CoV-2 virus has affected millions of people. However, it was noticed that high mortality was often a result of bacterial co-infections. One of the main pathogens responsible for secondary infections in patients with viral respiratory tract infections, including COVID-19, is *Staphylococcus aureus*. In recent years, the number of infections caused by drug-resistant strains of *S. aureus* has been growing rapidly, often exceeding the number of infections caused by antibiotic-sensitive strains. In addition, biofilm-related infections are more difficult to treat due to the lower sensitivity of biofilm structure to antibiotics. Bacteriophages are seen as alternative treatment of bacterial infections. Therefore, in our work, we have analyzed the efficacy of three Kayviruses against *S. aureus* strains isolated from COVID-19 patients. **Methods:** We analyzed the ability of tested phages to remove *S. aureus* biofilm both from polystyrene plates as well as from the surface of pulmonary epithelial cells. **Results:** We have observed that tested Kayviruses had a broad host range. Furthermore, phages were able to effectively reduce biofilm biomass and number of viable cells in pure culture. During our research, none of the tested phages was shown to have a negative effect on cell viability and were able to inhibit the negative effect *S. aureus* had on cell condition. **Conclusions:** Our results show tested phages were effective in reducing the biofilm of *S. aureus* strains isolated from COVID-19 patients, had no adverse effect on lung epithelial cell viability. Therefore, it should be recognized that the properties of three studied Kayviruses give them an advantage in the selection of phages for treatment of staphylococcal infections.

## 1. Introduction

COVID-19 has affected a large number of patients, with many being admitted to intensive care units and exposed to the discomfort of artificial ventilation. However, what was observed is that high mortality observed among artificially ventilated patients often resulted from secondary bacterial infection, rather than the primary viral one [1]. Due to the high incidence of infections, high infection rate with a severe course, and the severity of post-infection complications, particular interest should be paid to the co-infection with *Staphylococcus aureus* as it is one of the most commonly identified pathogens in COVID-19 patients [2,3]. *S. aureus* can asymptomatically colonize the respiratory tract, as well as cause infections of varying severity from local and relatively mild skin infections to life-threatening, including sepsis, infective endocarditis, and necrotizing pneumonia [4,5].

In addition to diseases related to the production of exotoxins and specific enzymes, *S. aureus* is known to cause biofilm-related infections, which are associated with diseases, such as osteomyelitis, periodontitis, endocarditis, chronic wound infection, and infection of grafts or prosthetics [6] *S. aureus* strains are known to easily acquire antibiotic resistance. In recent years, the number of infections caused by multidrug-resistant strains of *S. aureus* (MDRSA) in hospitals around the world has been growing rapidly, often exceeding the number of infections caused by antibiotic-sensitive strains [5,7]. In hospitalized COVID-19 patients, the treatment protocol for *S. aureus* pneumonia includes treatment with linezolid or vancomycin, followed by switching to penicillins (e.g., oxacillin) or cephalosporins if the MRSA phenotype is excluded [8]. It sometimes happens that antibiotics are not effective due to the biofilm formed on tissues of the respiratory tract, which is many times less sensitive to antibiotic treatment [9].

In response to the increase in bacterial resistance to antibiotics, bacteriophages are seen as one of the main candidates to be used in the treatment of bacterial infections. Their effectiveness on bacteria is constantly being tested by various scientific teams in terms of their impact on planktonic cells and bacterial biofilm. It is known that phages can prevent biofilm formation and maturation by destroying bacteria in the outer layer of the biofilm and inactivating planktonic cells. They can also penetrate existing biofilms and eliminate biofilm structure due to the phage-lysing enzymes, depolymerases, and lysine, released from cells along the progeny phages [10].

For a long time, it was believed that phages are neutral to eukaryotic cells and therefore safe to use in the treatment of higher vertebrates, including humans. However, the safety of treatment still requires analysis in various research models as new information on phage-cell interactions comes to light [11,12]. Therefore, in our work, we have analyzed the efficacy of three Kayviruses against *S. aureus* strains isolated from COVID-19 patients.

Bacteriophages belonging to the *Kayvirus* genus (named after their most known member Staphyloccoccal phage K) are a group of lytic phages infecting mainly *S. aureus* [13,14]. They are characterized by their broad host range, have documented efficacy against clinical isolates of this pathogen, and are known to be the main components of anti-Staphylococcal phage cocktails [15,16,17].

In this work, we have analyzed the ability of tested phages to remove *S. aureus* biofilm both from polystyrene plates as well as from the surface of pulmonary epithelial cells. We have also analyzed if phage treatment would influence viability of human cells in order to assess their safety as potential antibacterial agents.

## 2. Results

### 2.1. Phage Host Range Analysis

The first step in testing the efficacy of bacteriophages against *S. aureus* strains isolated from COVID-19 patients was host range and efficacy of plating analysis. We have observed that phage vB_SauM-A was able to infect 12 out of 26 tested strains, while phages vB_SauM-C and vB_SauM-D—15 strains, with 10 S. aureus strains serving as a host for all three phages (strains no. 5, 6, 9, 10, 11, 15, 17, 19, 21, 22). Only strains no. 1, 7, 8, 12, 14, 16, 24, and 25 were identified as resistant to infection with each of the tested phages (Table 1). The efficacy of plating (EOP) analysis showed that phage vB_SauM-A could be the most promising, as the EOP exceeded 100% in comparison to the primary host strain on 10 out of 12 tested hosts. For phage vB_SauM-C 4 strains were classified as good hosts, 9 strains as poor hosts, and 1 strain (no. 2) as very poor hosts with EOP ~0.2%. The least favorable results were obtained for phage vB_SauM-D with the majority of strains identified as poor or very poor hosts and only 3 strains classified as potentially good hosts (Table 2).

### 2.2. Biofilm Eradication by Phages

For the analysis of phage efficacy against biofilm formed by *S. aureus* strains from COVID-19 patients we have chosen 10 out of 26 strains (strains no. 5, 6, 9, 10, 11, 15, 17, 19, 21, 22), that were infected by all three tested Kayviruses. Then we have analyzed the influence of phage treatment on biofilm biomass, metabolic activity, and number of living cells (CFU/mL).

#### 2.2.1. Biofilm Biomass Assessment Using Crystal Violet Staining

**Figure 1:**
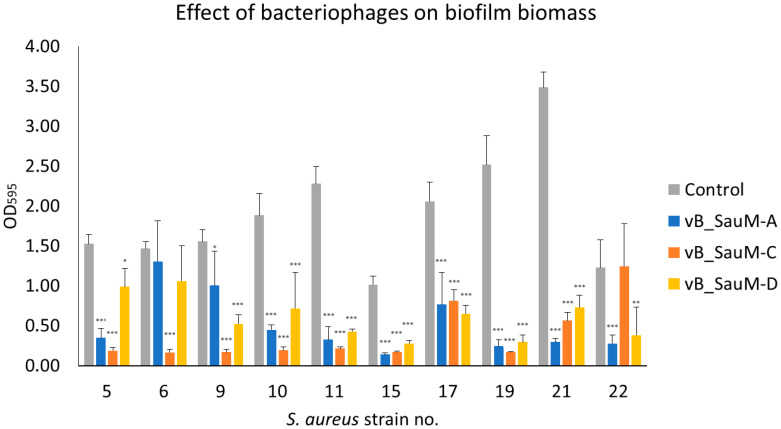
Reduction of *S. aureus* biofilm biomass by phages vB_SauM-A, vB_SauM-C and vB_SauM-D. Arithmetic mean of triplicates with error bars representing SD. Statistical analysis was performed using the Kruskal–Wallis test followed by Dunn’s multiple comparison test for values with nonparametric distribution, with * *p* < 0.05, ** *p* < 0.01, and *** *p* < 0.001.

The results of crystal violet staining showed that in the majority of tested variations, there was a clear reduction in biofilm biomass following phage inoculation compared to the non-treated wells. The most significant reduction was observed in the case of strain no. 10, 11, 15, 19, and 21 for all three phages, while least effective against strain no. 17. However, the result was still statistically significant. No significant differences in biomass reduction were observed for strain no. 5 treated with phage vB_SauM-D, strain no. 6 treated with vB_SauM-A and vB_SauM-C, and strain no. 22 treated with vB_SauM-C (Figure 1).

#### 2.2.2. Assessment of Biofilm Metabolic Activity

**Figure 2:**
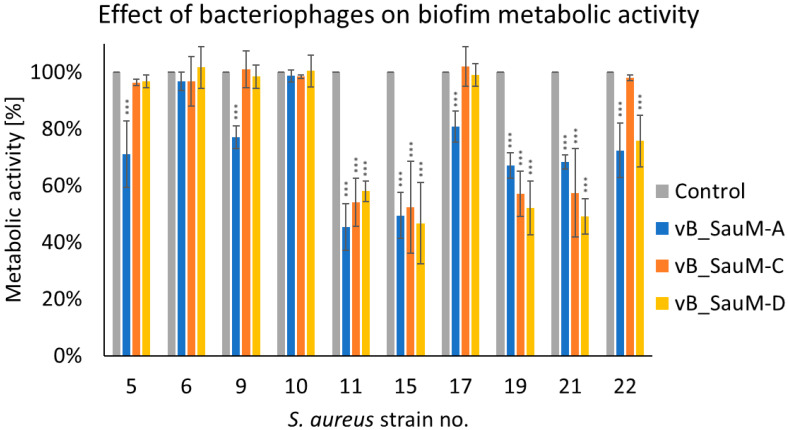
Influence of phages vB_SauM-A, vB_SauM-C, and vB_SauM-D on biofilm metabolic activity. Arithmetic mean of triplicates with error bars representing SD. Statistical analysis was performed using the Kruskal–Wallis test followed by Dunn’s multiple comparison test for values with nonparametric distribution, with *** *p* < 0.001.

Since crystal violet alone is currently thought to be insufficient to evaluate biofilm formation and antibiofilm properties of antimicrobial agents in order to obtain more information on phage activity against tested *S. aureus* strains we have additionally assessed biofilm metabolic activity [16,17]. While analyzing the metabolic activity of biofilm treated by phages we have observed that a significant decrease occurred for strains no. 11, 15, 19, and 21 in case of treatment with all three Kayviruses. For strain no. 22 a significant difference between treated samples and untreated control was observed in the case of phages vB_SauM-A and vB_SauM-D, while for strains no. 5, 9, and 17 only in the case of phage vB_SauM-A treatment (Figure 2).

#### 2.2.3. Assessment of Number of Viable Cells in Biofilm Using CFU/mL Count

**Figure 3:**
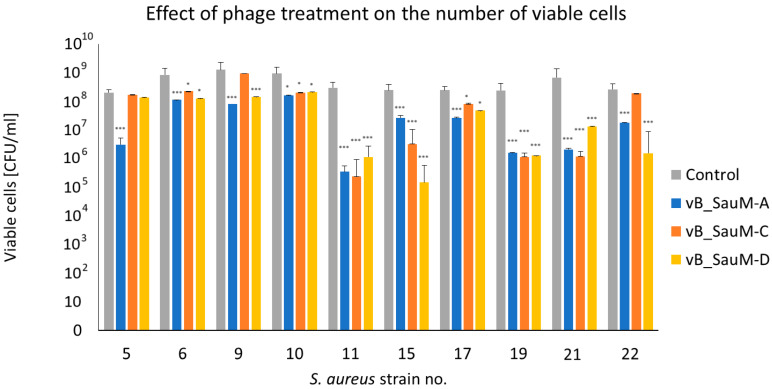
Quantitative analysis of in vitro anti-biofilm activity of phages vB_SauM-A, vB_SauM-C, and vB_SauM-D on *S. aureus* biofilm by CFU/mL count. Arithmetic mean of triplicates with error bars representing SD. Statistical analysis was performed using the Kruskal–Wallis test followed by Dunn’s multiple comparison test for values with nonparametric distribution, with * *p* < 0.05 and *** *p* < 0.001.

The assessment of the number of viable cells using CFU/mL estimation showed that in most cases the treatment of biofilm with one of three tested Kayviruses resulted in a statistically significant reduction in bacterial titer. The most significant decrease in CFU/mL (reaching ~2 log difference in comparison with untreated control) was observed in the case of strains no 11, 15, 19, and 21. However, no significant decrease in titer was observed in case of strain no. 5 treated with phages vB_SauM-C and vB_SauM-D, strains no. 9 and 22 treated with phage vB_SauM-C (Figure 3).

### 2.3. Analysis of Phage Treatment Efficacy of Infected Human Cell Line Using CFU/mL Count

**Figure 4:**
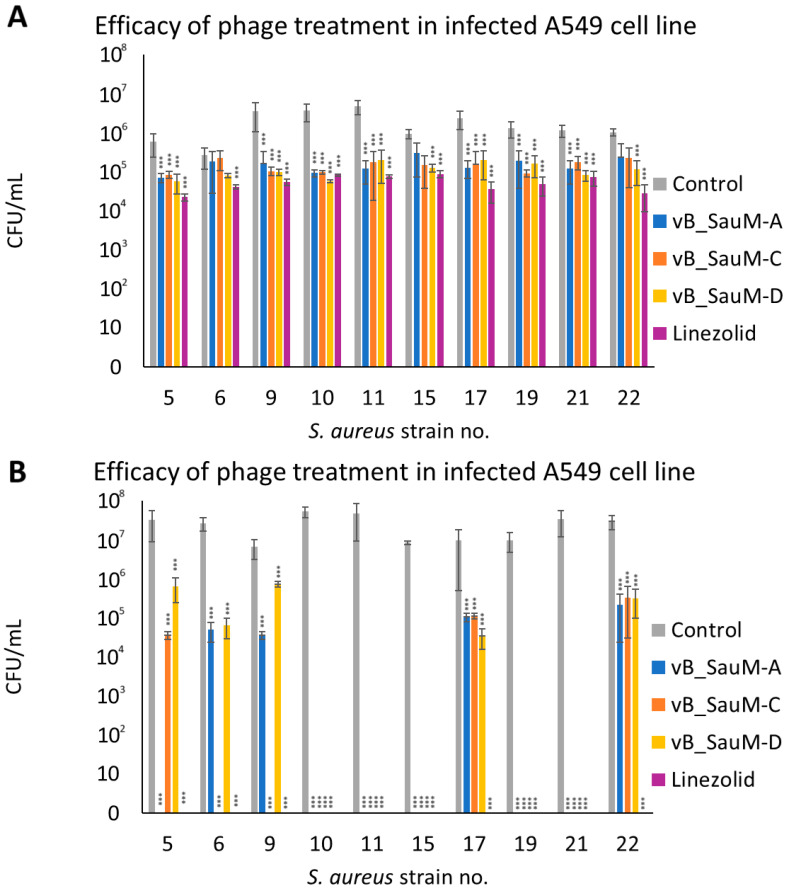
The CFU/mL of *S. aureus* strains isolated from COVID-19 patients in infected A549 cell line after treatment with phages vB_SauM-A, vB_SauM-C and vB_SauM-D or linezolid 2 h (**A**) and 4 h (**B**) post-exposure. Arithmetic mean of triplicates with error bars representing SD. Statistical analysis was performed using the Kruskal–Wallis test followed by Dunn’s multiple comparison test for values with nonparametric distribution, with *** *p* < 0.001.

The next step in our research was to analyze if phages were able to effectively reduce the number of bacteria from the surface of human cells (pulmonary epithelial cell line A549) and to compare it with the efficacy of linezolid in order to better evaluate phage therapeutic potential. After 2 h post-treatment, bacterial titer was significantly lower in the majority of tested combinations in comparison with the control, with the exception of strains no. 6, 15, and 22 treated with phages vB_SauM-A and vB_SauM-C (Figure 4A). After 4 h post-treatment, we observed that for strains 10, 11, 15, 19, and 21 all tested phages were able to reduce the number of viable bacterial cells below the detectable level (~100 CFU/mL), which corresponded with the efficacy of linezolid. In the case of strain no. 5 only phage vB_SauM-A had equal efficacy to linezolid, while phage vB_SauM-C had the same efficacy in the case of strains no. 6 and 9. In other tested variants the bacteria were detectable in the cell culture. However, the reduction of bacterial titer in comparison with untreated control was still deemed statistically significant (Figure 4B).

### 2.4. Analysis of Phage Treatment Influence on Viability of Human Cells Using Neutral Red Staining

**Figure 5:**
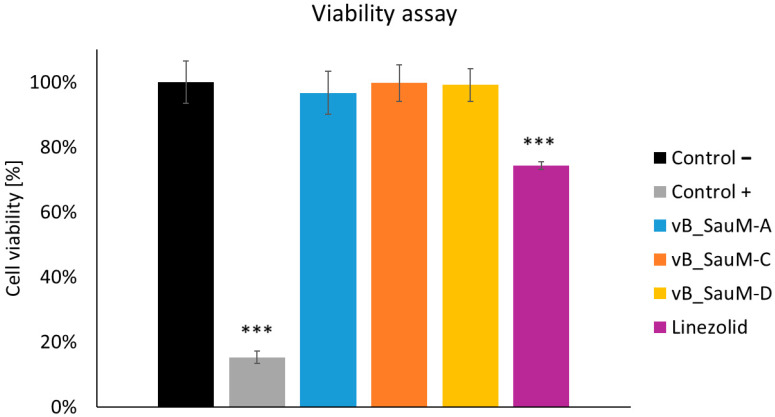
Cell viability of the A549 cells treated with phages vB_SauM-A, vB_SauM-C, vB_SauM-D, and linezolid in comparison to the untreated negative control and 10% DMSO-treated positive control. Arithmetic mean of triplicates with error bars representing SD. Statistical analysis was performed using the Kruskal–Wallis test followed by Dunn’s multiple comparison test for values with nonparametric distribution, with *** *p* < 0.001.

The last part of our study was the analysis of phage treatment influence on the viability of human pulmonary epidermal cells to evaluate the safety of the treatment. First, the effect of phages and linezolid on uninfected human cells was analyzed. We have observed no negative influence of phages on A549 cell line viability. However, linezolid in the concentration of 2 mg/mL did have a negative effect on cell viability (decrease to ~72%) (Figure 5).

**Figure 6:**
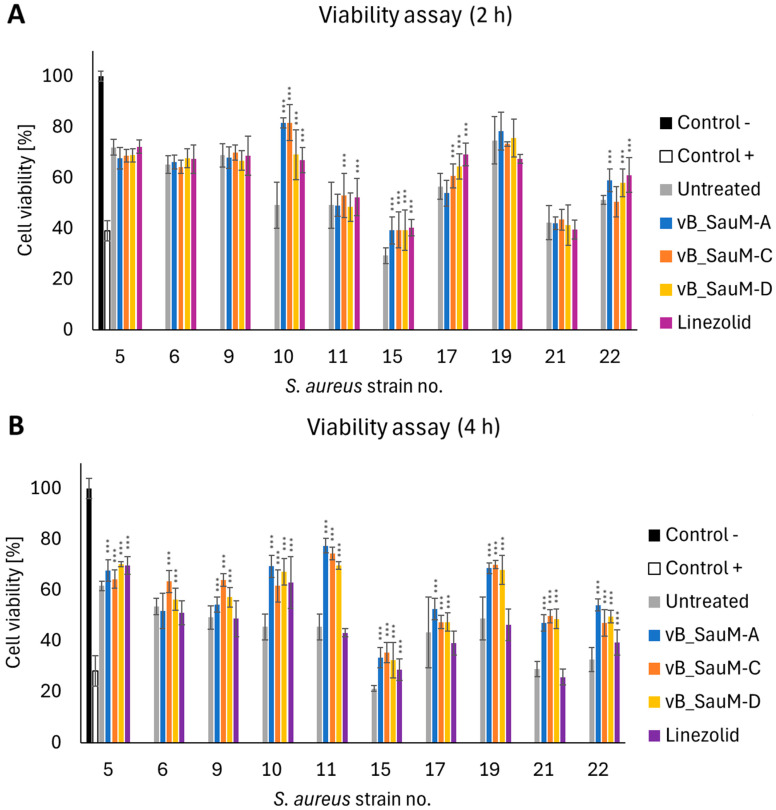
Viability of the A549 cells infected with *S. aureus* isolated from COVID-19 patients treated with phages vB_SauM-A, vB_SauM-C, vB_SauM-D, and linezolid after 2 h (**A**) and 4 h (**B**) post-exposure in comparison to the untreated control. Uninfected cells (negative control) and 10% DMSO-treated positive control are shown as reference values. Arithmetic mean of triplicates with error bars representing SD. Statistical analysis was performed using the Kruskal–Wallis test followed by Dunn’s multiple comparison test for values with nonparametric distribution, with *** *p* < 0.001.

While analyzing the influence of phage treatment on call viability we have noticed inhibition of the toxic effect of some *S. aureus* strains: 10, 11 (for phages vB_SauM-A and vB_SauM-D), 15, 17, and 22. After 4 h post-treatment, the difference in the viability of phage-treated and untreated cells was statistically significant in the majority of tested variations. However, in the case of linezolid, a significant decrease in viability was still visible in 6 out of 10 strains (strains no. 6, 9, 11, 17, 19, 21) (Figure 6).

## 3. Discussion

Breakouts of epidemics and pandemics of viral respiratory tract diseases such as influenza, SARS-CoV-1, and 2 result in high mortality rates not only due to infection itself but also as a result of bacterial co-infections, mainly pneumonia and bloodstream infections [19,20,21,22,23]. One of the main pathogens responsible for secondary infections in patients with viral respiratory tract infections, including patients with COVID-19, is *Staphylococcus aureus* [24,25,26]. Currently, the treatment protocol for *S. aureus*-caused pneumonia includes treatment with linezolid or vancomycin and then switching to penicillins (like oxacillin) or cephalosporins, if MRSA phenotype is ruled out [8]. However, the use of β-lactams could be impaired in some patients due to allergies and hypersensitivity [27,28,29]. The use of vancomycin is also debatable as this antibiotic has been reported to have a toxic effect on human cells, as well as the emergence of resistant strains is observed [30,31,32]. Therefore, alternatives to antibiotics should be available for the treatment of patients with secondary bacterial infections, whether they are caused by antibiotic-resistant strains or not [33,34].

In our work, we have analyzed the efficacy of three Kayviruses against *S. aureus* strains isolated from COVID-19 patients. All three phages have already been tested against multidrug-resistant clinical isolates and have proven effective in vitro and on the *Galleria mellonella* model [35]. We have observed, that all three Kayviruses had a broad spectrum of activity against *S. aureus* strains isolated from COVID-19 patients, with phages vB_SauM-C and vB_SauM-D being able to effectively infect more than half of the isolates. This corresponds with our previous observations of those phages having a wide host range, making them good candidates for potential therapy.

In the next step of our work, we have focused on the phage’s ability to remove *S. aureus* biofilm both from polystyrene plates as well as from the surface of pulmonary epithelial cells. We have previously shown that *fnbA* and *fnbB* genes, encoding proteins that facilitate the binding to the surface of eukaryotic cells were especially prevalent in strains isolated from COVID-19 patients. They were also able to adhere to human cells more effectively than the strains from the control group [36]. Therefore, it was important to us to analyze, whether the phages could effectively eradicate bacteria from the culture. We have observed, that phages were able to effectively reduce biofilm biomass, metabolic activity, and a number of viable cells in pure culture grown on polystyrene plates. During the experiment with infected A549 culture, we observed that in the case of 5 out of 10 tested strains, all phages were as effective as linezolid, and reduced the number of viable *S. aureus* strains below the level of detection after 4 h of treatment. This corresponds with other studies showing that bacteriophages are able to effectively reduce the number of bacteria on the surface of human cells [37].

While analyzing phage therapy safety during the previous studies we observed, that phage vB_SauM-A in concentrations such as 10^9^ and 10^8^ PFU/mL had a minor, but statistically significant negative influence on human skin fibroblasts (BJ) (a drop in viability to ~96%) [38]. However, this effect was not observed for the A549 line. During our research, none of the tested phages was shown to have a negative effect on pulmonary cell viability in contrast to linezolid. Furthermore, we have also observed, that treatment with phages was able to inhibit the negative effect *S. aureus* had on cell viability 4 h post-treatment, similar to what was reported for the BJ cell line infected with multidrug-resistant strains while the cells treated with linezolid still expressed the decline in health [38]. This observation was important, especially in the case of *S. aureus* COVID-19 strain no. 9 which was identified as carrying the gene encoding *tst* gene, responsible for the production of staphylococcal Toxic Shock Syndrome Toxin-1 (TSST-1) [36], implying that the use of phage therapy had no influence on toxin release as it is sometimes raised as a concern in discussing phage therapy safety [39,40].

## 4. Materials and Methods

### 4.1. Staphylococcus Aureus Strains

*Staphylococcus aureus* strains were isolated from patients diagnosed with COVID-19 (SARS-CoV-2 positive test) who were admitted to the hospital in Kościerzyna (Poland). The strains have been previously characterized regarding the presence of virulence factors and pathogenic potential [36].

### 4.2. Bacteriophages

For the preparation of phage lysates, an overnight culture of host strain in liquid LB medium was added to fresh LB in a 1:100 ratio and incubated at 37 °C with 150 rpm agitation. When the OD_600_ reached 0.1 the bacteria were infected with phages at a multiplicity of infection (MOI) of 0.1 and incubated at 37 °C until lysis occurred. Polyethylene glycol (PEG) 8000 (BioShop, Burlington, ON, Canada) was then added (final concentration 10% *w*/*v*) and stirred overnight at 4 °C. The precipitate was collected by centrifugation at 10,000× *g* for 20 min at 4 °C and suspended in 10 mL of TM buffer (10 mM Tris-HCl, 10 mM MgSO_4_ [pH 7.2]). PEG8000 was removed by adding 1 mL of chloroform and centrifugation at 3000× *g* for 15 min. The procedure was repeated until no PEG8000 residue was observed after centrifugation. The lysates were enumerated using the double-layer agar method and stored at 4 °C.

### 4.3. Human Cell Line and Culture Conditions

Human carcinoma, lung epithelial cells line A549 (ATCC, CCL-185) (ATCC, Manassas, VA, USA) were cultured in 75 cm^2^ U-Shaped Canted Neck Cell Culture Flask (Corning, New York, NY, USA) in an F-12K culture medium (ATCC, Manassas, VA, USA) supplemented with 10% fetal bovine serum (FBS), at 37 °C in a humidified atmosphere of 95% air and 5% CO_2_ in the HeraCell 150 incubator (Heraeus, Hanau, Germany) in accordance with manufacturer guidelines.

### 4.4. Phage Host Range Analysis

The host range analysis of phages vB_SauM-A, vB_SauM-C, and vB_SauM-D was performed using the double-layer agar plate technique. Phage lysates were serially diluted in 0.9% NaCl. Then 2.5 μL of each dilution was spotted onto a double-layer agar plate containing different *S. aureus* strains and left to air dry. The plates were incubated overnight at 37 °C. Following the incubation the plaques that were formed in the bacterial lawn were counted and phage titer was calculated. The efficacy of plating was then established by calculating the ratio between the phage titer obtained on the tested strain to the titer on the control (host) strain.

### 4.5. Biofilm Eradication by Phages

Biofilms were formed on 24-well polystyrene microtiter plates using the protocol described before [41]. Each well was inoculated with 500 μL of bacterial suspension, and plates were then incubated for 24 h at 37 °C. After incubation, the medium was discarded and the biofilms were washed with 0.9% NaCl. Next, 500 μL of phage lysate (10^9^ PFU/mL) was added to the wells. LB medium was added instead of phage lysate in wells serving as a control variant. After incubation for 24 h at 37 °C, microplates were washed twice with 0.9% NaCl, and biofilm eradication was assessed with colony-forming unit (CFU) quantification, crystal violet staining, and metabolic activity.

#### 4.5.1. Biofilm Biomass Assessment Using Crystal Violet Staining

The crystal violet (CV) assay was applied as described previously [41]. Briefly, the attached bacteria were fixed with methanol and stained with 1% crystal violet for 15 min. The plates were then washed with distilled water to remove the excess stain and left to air dry. The CV stain was solubilized by adding 200 μL of ethanol-acetic acid-water (30:30:40) and the optical density at 595 nm was measured in the microplate spectrophotometer (BioTek EPOCH, BioTek Instruments, Winooski, VT, USA).

#### 4.5.2. Assessment of Biofilm Metabolic Activity

The assessment of biofilm metabolic activity was performed using BacTiter-Glo™ Microbial Cell Viability Assay (Promega, Madison, WA, USA) in accordance with manufacturer protocol. The results were presented as % of metabolic activity in comparison to untreated control (100%).

#### 4.5.3. Assessment of Number of Viable Cells in Biofilm Using CFU/mL Count

The number of bacteria adhered to the surface of microplate wells was enumerated as described by Kaźmierczak et al. [41]. Therefore, the plate contents were aspirated and washed twice with 500 μL of 0.9% NaCl. Subsequently, 500 μL of 0.9% NaCl was added to each well, and the biofilm was disrupted by vigorous pipetting. The 10-fold serial dilutions were immediately performed in 0.9% NaCl, and 40 μL of the dilutions were directly plated on LB plates. After 24 h of incubation, the colonies were counted to calculate the CFU/mL.

### 4.6. Analysis of Phage Treatment Efficacy of Infected Human Cell Lines Using CFU/mL Count

*S. aureus* suspension for cell infection was prepared as follows: an overnight culture of *S. aureus* was added to fresh medium at a 1:100 ratio and incubated with shaking (37 °C, 150 rpm) until OD_600_ = 0.1. One mL of culture was then centrifugated for 5 min at 4 °C and 1000× *g*. The supernatant was discarded and the pellet was washed twice with 0.9% NaCl. Bacteria were then suspended in 1 mL of fresh F-12K medium.

A549 cells were plated into 96-well tissue culture plates (Nest Scientific Biotechnology, Wuxi, China) at a density of 10^4^ per well and allowed to attach for 24 h in F-12K supplemented with 10% FBS. After 24 h, the medium was removed and the cells were infected with 10^4^ CFU/mL of *S. aureus*. After 2 h phage lysate was added to the final concentration of 10^9^ PFU/mL, and one set of wells was treated with linezolid (2 mg/mL). A set of untreated wells was used as a control. After 2 and 4 h of incubation, 50 μL of the medium was removed and serial dilutions were made in 0.9% NaCl. Fifty μL of each dilution was spread on LB-agar plates. The samples were then incubated overnight at 37 °C and colonies were counted to obtain CFU/mL.

### 4.7. Analysis of Phage Treatment Influence on Viability of Human Cells Using Neutral Red Staining

The assay was performed in accordance with the protocols published previously with some modifications [42]. The cells were treated as described in Section 4.6. Additionally, a set of cells was treated with 10% DMSO (Sigma-Aldrich, St. Louis, MO, USA)—as a positive control. Following incubation, the supernatant was discarded and replaced with 100 μL of F-12K with 0.33% neutral red (Sigma-Aldrich) (1:40 ratio). After 2 h of incubation at 37 °C, the medium was removed, and cells were washed with 100 μL of Phosphate-Buffered Saline (PBS) per well. Cells were then treated with 150 μL of a de-staining solution (50% ethanol, 49% distilled H_2_O, and 1% acetic acid [Alchem, Torun, Poland]) and incubated with shaking at 37 °C for 10 min. Absorbance was measured at 540 nm (SynergyH1, BioTek Instruments, Winooski, VT, USA).

### 4.8. Statistical Analysis

Experiments were carried out in triplicates to calculate means which were compared using the Kruskal–Wallis test followed by Dunn’s multiple comparison test for values with the nonparametric distribution. The Wilcoxon signed-rank test was used to assess if there was a significant difference between time points.

## Figures and Tables

**Table 1:** The titer of bacteriophages vB_SauM-A, vB_SauM-C, and vB_SauM-D on 26 *S. aureus* strains isolated from COVID-19 patients in comparison to isolation host (control) strain. Arithmetic mean of triplicates with SD.

*S. aureus* Strain	Mean Phage Titer and SD					
	vB_SauM-A		vB_SauM-C		vB_SauM-D	
Host strain	3.00 × 10^10^	-	2.00 × 10^10^	-	2.60 × 10^10^	-
COVID-19 1	-	-	-	-	-	-
COVID-19 2	-	-	4.00 × 10^7^	±1.74 × 10^7^	5.33 × 10^8^	±1.22 × 10^8^
COVID-19 3	-	-	-	-	5.60 × 10^7^	±4.21 × 10^7^
COVID-19 4	-	-	-	-	7.33 × 10^8^	±4.64 × 10^8^
COVID-19 5	4.48 × 10^11^	±2.95 × 10^11^	6.93 × 10^9^	±3.35 × 10^9^	1.76 × 10^9^	±1.18 × 10^9^
COVID-19 6	1.34 × 10^9^	±6.02 × 10^8^	1.39 × 10^9^	±9.10 × 10^8^	3.60 × 10^9^	±3.47 × 10
COVID-19 7	-	-	-	-	-	-
COVID-19 8	-	-	-	-	-	-
COVID-19 9	1.58 × 10^11^	±1.40 × 10^11^	2.93 × 10^9^	±1.97 × 10^9^	3.07 × 10^9^	±4.62 × 10^8^
COVID-19 10	1.53 × 10^11^	±1.48 × 10^11^	2.61 × 10^9^	±2.60 × 10^9^	4.31 × 10^9^	±3.28 × 10^9^
COVID-19 11	1.54 × 10^11^	±1.32 × 10^11^	3.07 × 10^9^	±4.62 × 10^8^	1.80 × 10^9^	±3.12 × 10^8^
COVID-19 12	-	-	-	-	-	-
COVID-19 13	-	-	1.47 × 10^9^	±8.33 × 10^8^	-	-
COVID-19 14	-	-	-	-	-	-
COVID-19 15	1.27 × 10^11^	±1.12 × 10^11^	4.95 × 10^8^	±4.72 × 10^8^	1.48 × 10^9^	±1.86 × 10^9^
COVID-19 16	-	-	-	-	-	-
COVID-19 17	7.69 × 10^10^	±9.27 × 10^10^	9.67 × 10^8^	±	-	-
COVID-19 18	8.41 × 10^10^	±7.39 × 10^10^	-	-	1.43 × 10^9^	±1.58 × 10^9^
COVID-19 19	9.35 × 10^10^	±8.22 × 10^10^	9.55 × 10^8^	±1.60 × 10^9^	1.01 × 10^9^	±1.21 × 10^9^
COVID-19 20	1.12 × 10^9^	±5.25 × 10^8^	-	-	-	-
COVID-19 21	5.61 × 10^10^	±5.09 × 10^10^	4.93 × 10^8^	±2.60 × 10^8^	6.00 × 10^8^	±2.40 × 10^8^
COVID-19 22	1.44 × 10^11^	±1.24 × 10^11^	6.53 × 10^8^	±2.44 × 10^8^	1.05 × 10^9^	±3.72 × 10^8^
COVID-19 23	-	-	4.59 × 10^8^	±7.46 × 10^8^	3.17 × 10^8^	±1.09 × 10^8^
COVID-19 24	-	-	-	-	-	-
COVID-19 25	-	-	-	-	-	-
COVID-19 26	-	-	4.79 × 10^8^	±7.29 × 10^8^	-	-

**Table 2:** The efficacy of plating (EOP) of phages vB_SauM-A, vB_SauM-C, and vB_SauM-D on *S. aureus* isolated from COVID -19 patients described as % of titer obtained on isolation host strain. Arithmetic mean of triplicates with SD. EOP ≥ 100% very good host, 99–10% good host, 9.9–0.1% poor host, ≤0.1% very poor host [18].

*S. aureus* Strain	EOP with SD		
	vB_SauM-A	vB_SauM-C	vB_SauM-D
Host strain	100%	100%	100%
COVID-19 2	-	0.20 ± 0.09%	2.05 ± 0.47%
COVID-19 3	-	-	0.22 ± 0.16%
COVID-19 4	-	-	2.82 ± 1.78%
COVID-19 5	1493.33 ± 66.85%	34.67 ± 16.77%	6.77 ± 4.53%
COVID-19 6	4.47 ± 0.013%	6.93 ± 4.55%	13.85 ± 3.35%
COVID-19 9	525.33 ± 31.36%	14.67 ± 9.87%	11.79 ± 1.78%
COVID-19 10	511.33 ± 33.08%	13.07 ± 12.99%	16.56 ± 2.63%
COVID-19 11	512.69 ± 29.46%	15.33 ± 2.31%	6.92 ± 1.20%
COVID-19 13	-	7.33 ± 4.16%	-
COVID-19 15	423.22 ± 20.05%	2.47 ± 1.36%	5.68 ± 0.17%
COVID-19 17	256.20 ± 20.69%	4.83 ± 0.94%	-
COVID-19 18	280.33 ± 16.50%	-	5.49 ± 6.08%
COVID-19 19	311.56 ± 18.34%	4.77 ± 0.99%	3.89 ± 0.66%
COVID-19 20	3.73 ± 0.12%	-	-
COVID-19 21	186.91 ± 11.27%	2.47 ± 1.30%	2.31 ± 0.92%
COVID-19 22	481.58 ± 27.71%	3.27 ± 1.22%	4.05 ± 1.43%
COVID-19 23	-	2.29 ± 0.73%	1.22 ± 0.42%
COVID-19 26	-	2.39 ± 0.64%	-

## Data Availability

Raw data is available per request from the authors.
